# A new class of organogelators based on triphenylmethyl derivatives of primary alcohols: hydrophobic interactions alone can mediate gelation

**DOI:** 10.3762/bjoc.13.17

**Published:** 2017-01-23

**Authors:** Wangkhem P Singh, Rajkumar S Singh

**Affiliations:** 1 Organic Materials Research Laboratory, Department of Basic Sciences & Social Sciences, North-Eastern Hill University, Shillong-793022, Meghalaya, India

**Keywords:** hydrophobic interactions, organogelator, SEM, triphenylmethyl group, xerogel

## Abstract

In the present work, we have explored the use of the triphenylmethyl group, a commonly used protecting group for primary alcohols as a gelling structural component in the design of molecular gelators. We synthesized a small library of triphenylmethyl derivatives of simple primary alcohols and studied their gelation properties in different solvents. Gelation efficiency for some of the derivatives was moderate to excellent with a minimum gelation concentration ranging between 0.5–4.0% w/v and a gel–sol transition temperature range of 31–75 °C. 1,8-Bis(trityloxy)octane, the ditrityl derivative of 1,8-octanediol was the most efficient organogelator. Detailed characterizations of the gel were carried out using scanning electron microscopy, FTIR spectroscopy, rheology and powder XRD techniques. This gel also showed a good absorption profile for a water soluble dye. Given the non-polar nature of this molecule, gel formation is likely to be mediated by hydrophobic interactions between the triphenylmethyl moieties and alkyl chains. Possible self-assembled packing arrangements in the gel state for 1,8-bis(trityloxy)octane and (hexadecyloxymethanetriyl)tribenzene are presented. Results from this study strongly indicate that triphenylmethyl group is a promising gelling structural unit which may be further exploited in the design of small molecule based gelators.

## Introduction

Small organic molecules capable of forming gels are called low molecular weight gelators (LMWGs) [1–3]. These LMWGs can immobilize organic solvents (forming organogels) and water or aqueous solvents (forming hydrogels) under different experimental conditions. Gels, so formed are supramolecular in nature as they result from self-assembly of the gelator molecules through secondary interactions like H-bonding, π-stacking, donor–acceptor interaction, electrostatic, metal coordination, hydrophobic forces or van der Waals’ interactions [4–6]. We know a great deal about the aggregation behaviour of gelator molecules from many studies conducted during the past several years. However, rational design and accurate prediction of the gel forming ability of a given molecule is still a formidable challenge, if not altogether impossible [7–8]. Often, the discovery of new gelators relies on an empirical approach wherein structural components capable of forming non-covalent interactions are incorporated into different molecular scaffolds and tested for their gelation abilities. In another approach, modifications are made to known gelator scaffolds in the search for new gelators. These approaches have been used widely with a fair degree of success giving rise to gelator molecules with diverse molecular structures [9–13]. They are being increasingly explored for a variety of uses in the fields of materials science, biomedicines, environment science, etc. Hence, the discovery of new functional gelators continues to be an important focus of research worldwide.

Since most gels are formed by non-covalent interactions, they can be assembled or disassembled in response to appropriate external stimuli. Functional gels have been reported that are sensitive to physical stimuli like UV–vis light [14], ultrasound [15], and mechanical forces [16]. On the other hand, chemical stimuli-sensitive gels respond to stimuli such as acids or bases [17], metal ions [18], oxidation and reduction [19], reactive species enzymes, etc. Such responsive gel systems are highly desirable in stimuli-responsive sensor materials, drug delivery, catalysis, nano- and mesoscopic assemblies, light harvesting systems, and many others [20–22].

As mentioned above, developing a new gelator is still largely a trial and error method. The most widely used design strategy involves the inclusion of structural components favouring intermolecular non-covalent interactions (like H-bonding, π-stacking, donor–acceptor interaction, metal coordination, ionic, hydrophobic forces, etc.) into the gelator molecular structure and studying their gelation behaviour. H-bonding interaction (present in amide, urea, carbamate linkages, etc) alone or in combination with other interactions is the most extensively used strategy in the design of gelators [23–24]. Similar is the case for π-stacking interaction (seen in naphthalene, anthracene, pyrene, perylene, etc.) which has been mostly used in combination with other types of secondary interactions in the discovery of many gelators [25–26]. This holds true for many other non-covalent interactions as well. As a consequence of this approach, numerous gelator molecules with highly varied structures have been reported. Because of the structural diversity, gelation behaviours also vary significantly. Taken together, the development of new gelators using this empirical approach still continues to be quite successful. Interestingly, we observed that the design of gelators based on hydrophobic interactions alone (not in combination with other types of secondary interactions) is underexplored compared to the overwhelming use of other types of interactions [27–28]. Hence, we were motivated to search for new hydrophobic structural components which can be incorporated in the design of new gelators. Another important aspect we also considered is the accessibility of gelator molecules in large quantities from cheap starting materials in as few synthetic steps as possible. Keeping these two aspects in mind, we explored and identified the triphenylmethyl group [29], a commonly used protecting group for primary alcohols as a potential gelling structural component in the design of new gelators. To the best of our knowledge, the use of the triphenylmethyl (trityl) group as a potential gelling structural component in the design of molecular gelators has not been reported before. We reasoned that the triphenylmethyl group (having three benzene rings) will be a good candidate for hydrophobic interactions mediated gelation. We also noted that since the triphenylmethyl group as a whole exhibits a non-planar geometry (unlike the planar geometry of say anthracene, pyrene, etc.), π–π stacking will be favoured only between benzene units of different trityl groups. A small library of triphenylmethyl derivatives was synthesized from the corresponding primary alcohols employing a single step reaction and detailed gelation studies carried out. Remarkably, we found that some of these triphenylmethyl derivatives can act as efficient gelators of some polar solvents thereby validating our approach.

## Results and Discussion

### Synthesis

**Scheme 1:**
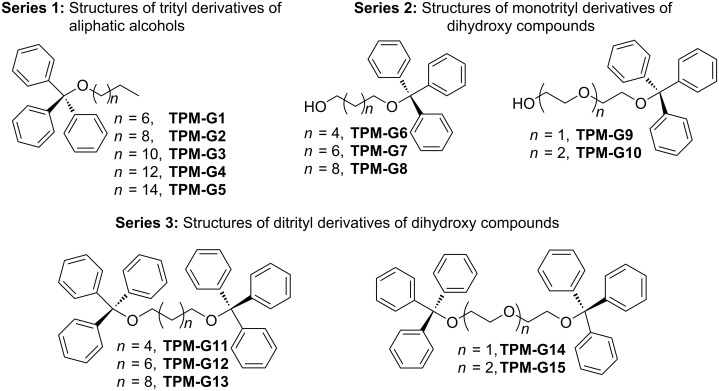
Chemical structures of triphenylmethyl-based organogelators.

We synthesized a small library of triphenylmethyl derivatives of easily available simple primary alcohols (Scheme 1, **TPM-G1**–**TPM-G15**). Trityl derivatives of simple aliphatic alcohols afforded the Series 1 compounds. Simple dihydroxy compounds can generate both mono-trityl (Series 2) and ditrityl derivatives (Series 3). The mono-trityl derivatives were synthesized in one step by the reaction of the corresponding alcohols (1 equivalent) with trityl chloride (1 equivalent) in the presence of triethylamine (1 equivalent) in dichloromethane (DCM) at room temperature (Scheme 1, **TPM-G1**–**TPM-G10**). On using two equivalents of trityl chloride and triethylamine with one equivalent of the dihydroxy compounds, the respective ditrityl derivatives were obtained (Scheme 1, **TPM-G11**–**TPM-G15**).

### Gelation behaviour

**Figure 1:**
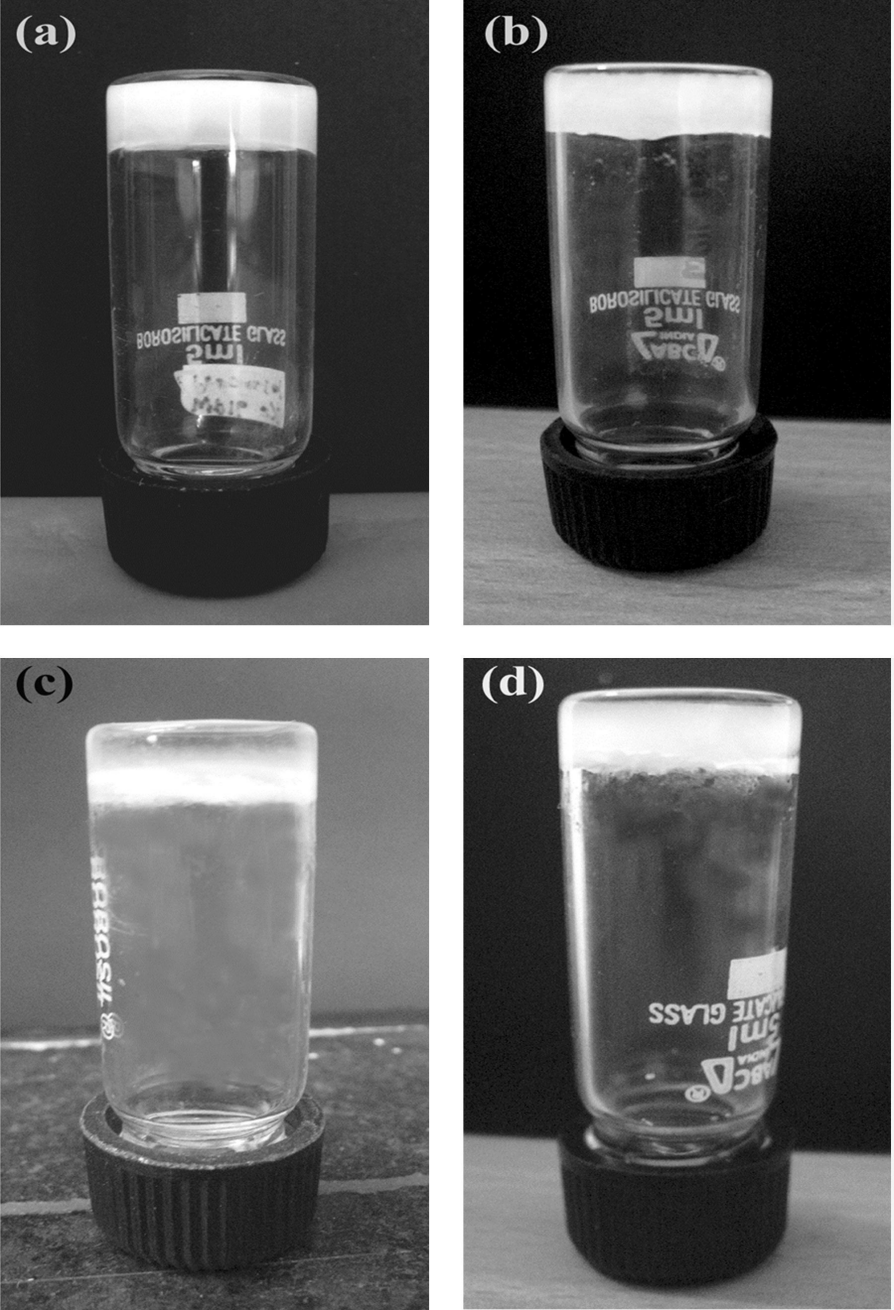
Organogels formed by **TPM-G12** in (a) propan-1-ol; (b) DMSO at 0.5% w/v; (c) organogel from **TPM-G5** in DMSO at 2% w/v; (d) organogel from **TPM-G4** in diethylene glycol at 4% w/v.

We evaluated the gelation behaviour of the 15 triphenylmethyl derivatives in 21 different organic solvents (both polar and non-polar solvents) at a concentration of 2% w/v. The gelation behaviour is summarized in Table 1. Three of the compounds (**TPM-G4**, **TPM-G5**, **TPM-G12**) formed organogels in polar solvents like dimethylsulfoxide (DMSO), propan-1-ol, propan-2-ol, butan-1-ol, butan-2-ol, diethylene glycol, and triethylene glycol. **TPM-G5** (trityl derivative of 1-hexadecanol) formed an organogel in DMSO while **TPM-G4** (trityl derivative of 1-tetradecanol) was able to gel diethylene glycol and triethylene glycol (Figure 1). **TPM-G12** (the ditrityl derivative of 1,8-octanediol) turned out to be an excellent gelator of some polar solvents. It formed gels in DMSO, propan-1-ol, propan-2-ol, butan-1-ol, and butan-2-ol (Figure 1). The gels formed are opaque and not transparent or translucent. Since these triphenylmethyl derivatives are non-polar, the formation of gels in polar solvents (and not in non-polar solvents) is understandable. Gel formation in polar solvents will be driven mostly by hydrophobic interactions between the alkyl chains and the triphenylmethyl moieties. Compared to other planar aromatic molecules (such as naphthalene, anthracene, perylene, etc.), the triphenylmethyl moiety as a whole exhibits a non-planar geometry, hence π-stacking interaction between triphenylmethyl moieties may not be optimal. The π-stacking interaction may, however, be present between the benzene units of different triphenylmethyl moieties. It is interesting to note here that the presence of hydrophobic interactions alone (in the absence of H-bond forming structural components) is sufficient for gel formation in polar solvents. For Series 1 compounds, the presence of longer alkyl chains (tetradecyl and hexadecyl) favoured the formation of gels. None of the Series 2 derivatives, containing one hydroxy group and one triphenylmethyl group, formed gels in the solvents tested. The contrasting gelation behaviour of **TPM-G1** (non-gelator), **TPM-G7** (non-gelator), and **TPM-G12** (gelator) is worth highlighting. **TPM-G1** contains a triphenylmethyl moiety at one end of an octyl chain, **TPM-G7** has a triphenylmethyl moiety and a hydroxy group at the two ends of an octyl chain, whereas **TPM-G12** has two triphenylmethyl moieties at the two ends of an octyl chain. The presence of an additional triphenylmethyl group (in **TPM-G12**, and not in **TPM-G1** and **TPM-G7**) has a profound effect on gelation behaviour as shown in Table 1 and Figure 1. Although **TPM-G12** and **TPM-G15** (with three oxyethylene units flanked by two triphenylmethyl groups) are of similar molecular size and shape, **TPM-G15** did not form a gel in any solvents. The presence of the more polar oxyethylene unit (compared to non-polar octyl chain) had a negative effect on the gelation behaviour of **TPM-G15**. **TPM-G11** (the ditrityl derivative of 1,6-hexanediol) and **TPM-G13** (the ditrityl derivative of 1,10-decanediol) did not form a gel implying that an octyl chain is the optimal length for gel formation.

**Table 1:** Gelation properties of **TPM-G1 – TPM-G15**^a,b^.

Solvent	**TPM** **-G1**	**TPM** **-G2**	**TPM** **-G3**	**TPM** **-G4**	**TPM** **-G5**	**TPM** **-G6**	**TPM** **-G7**	**TPM** **-G8**	**TPM** **-G9**	**TPM** **-G10**	**TPM** **-G11**	**TPM** **-G12**	**TPM** **-G13**	**TPM** **-G14**	**TPM** **-G15**

benzene	S	S	S	S	P	S	S	S	S	S	S	S	S	S	S
toluene	S	S	S	S	S	S	S	S	S	S	S	S	S	S	S
o-xylene	S	S	S	S	S	S	S	S	S	S	S	S	S	S	S
nitrobenzene	S	S	S	S	S	S	S	S	S	S	S	S	S	S	S
chlorobenzene	S	S	S	S	P	S	S	S	S	S	S	S	S	S	S
1,2-dichlorobenzene	S	S	S	S	S	S	S	S	S	S	S	S	S	S	S
cyclohexane	S	S	S	S	S	S	S	S	S	S	S	S	S	S	S
THF	S	S	S	S	S	S	S	S	S	S	S	S	S	S	S
DMF	S	S	S	S	S	S	S	S	S	S	S	S	S	S	S
acetonitrile	S	S	S	S	T	S	S	S	P	S	I	I	S	I	S
1,4-dioxane	S	S	S	S	S	S	S	S	S	S	S	S	S	S	S
DMSO	S	S	T	P	**G**(2.0)	S	S	S	S	S	T	**G**(0.5)	S	S	S
methanol	S	S	S	P	P	S	S	S	S	S	I	I	I	P	I
propan-1-ol	S	S	S	S	S	S	S	S	S	S	I	**G**(0.5)	S	P	S
propan-2-ol	S	S	S	S	S	S	S	S	S	S	I	**G**(0.5)	S	P	S
butan-1-ol	S	S	S	S	S	S	S	S	S	S	P	**G**(0.6)	S	S	S
butan-2-ol	S	S	S	S	S	S	S	S	S	S	I	**G**(0.6)	S	P	S
ethyleneglycol	S	T	T	T	S	T	T	T	S	T	S	T	S	P	S
diethyleneglycol	T	T	T	**G**(4.0)	T	S	T	S	S	S	S	S	T	P	S
triethyleneglycol	S	S	T	**G**(8.0)	T	S	S	S	S	S	S	S	P	P	S
benzylalcohol	S	S	S	S	S	S	S	S	S	S	S	S	S	S	S

^a^Values given inside the brackets denote the minimum gel concentration (MGC, % w/v ) to form organogels at room temperature. G: gel; S: solution; P: precipitate; I: insoluble; T: turbid. ^b^THF, DMSO, and DMF indicate tetrahydrofuran, dimethyl sulfoxide and *N*,*N*-dimethylformamide, respectively.

**Figure 2:**
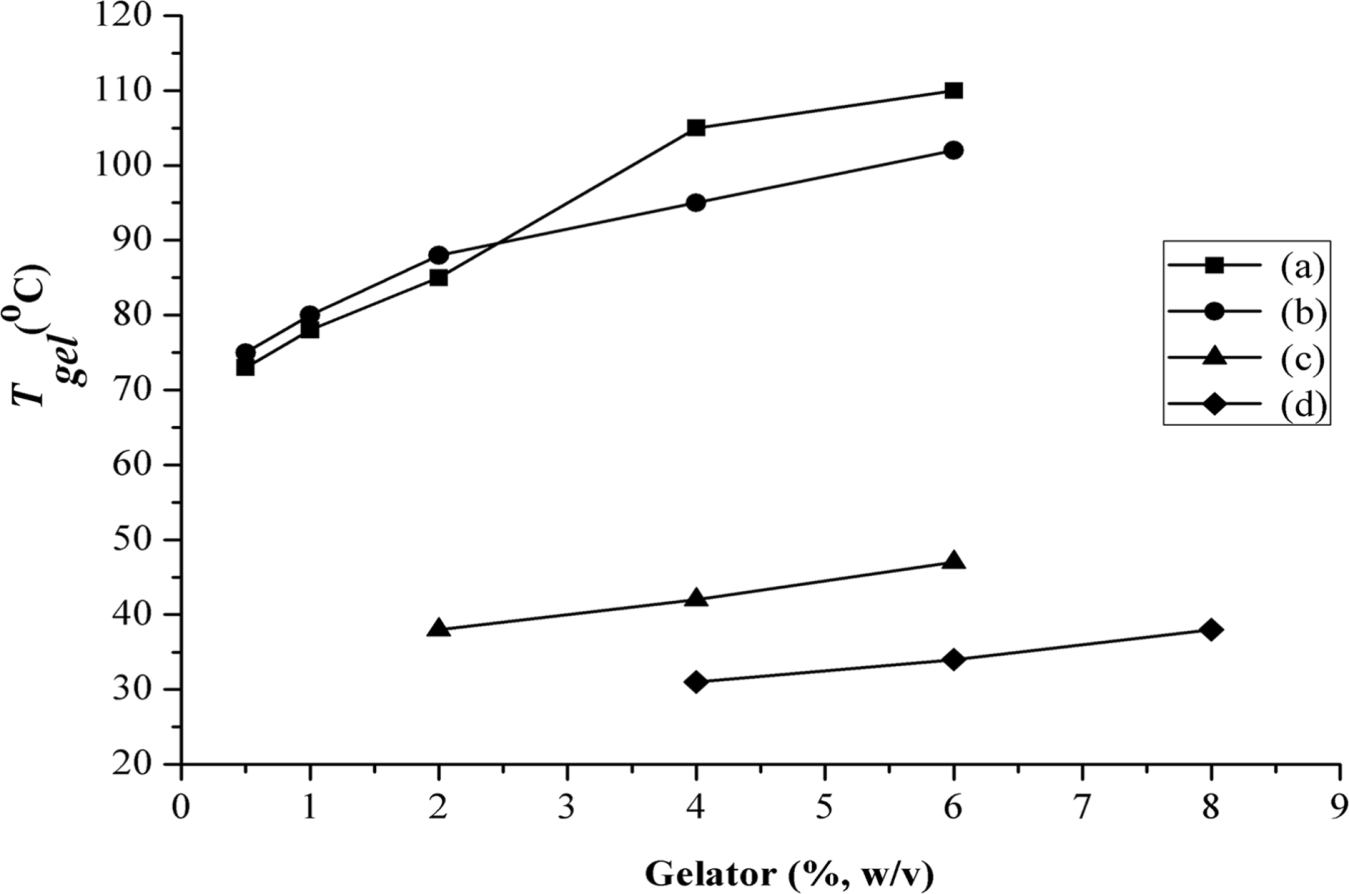
Plot of *T*_gel_ (gel–sol transition temperature) versus gelators at different concentrations. **TPM-G12** in (a) DMSO and (b) propan-1-ol; (c) **TPM-G5** in DMSO; (d) **TPM-G4** in diethylene glycol.

The minimum gelation concentration (MGC) of the three gelators were determined [30]. They are in the range of 0.5–4.0% w/v (Table 1). **TPM-G12** is an excellent gelator with an MGC value of 0.5% in both propan-1-ol and DMSO. MGC values for **TPM-G5** and **TPM-G4** are 2.0% (DMSO) and 4.0% (diethyleneglycol), respectively. Figure 2 shows the gel–sol transition temperature (*T*_gel_) of the gels which were determined using the dropping ball method [31]. *T*_gel_ values (at MGC) for **TPM-G12** are 73 °C (DMSO) and 75 °C (propan-1-ol). *T*_gel_ values for **TPM-G5** and **TPM-G4** were much lower at 38 °C and 31 °C, respectively, at the MGC. As expected, with an increase in gelator concentration there is an increase in *T*_gel_ for all the three gelators.

### Stability of gels

The room temperature stability of the gels was also monitored. The organogel from **TPM-G12** (1% w/v in propan-1-ol and DMSO) remained stable for a long time (more than a month, Figure S4 in Supporting Information File 1) without any appreciable change in its structural integrity. However, on prolonged storage at room temperature, there is a gradual loss of the entrapped solvent from the gels. **TPM-G5** also formed a strong gel in DMSO which is stable at room temperature for a long time (≈2 weeks). In contrast, organogels formed from **TPM-G4** in diethylene glycol and triethylene glycol were weak and not stable at prolonged storage (≈2 days). This is reflected in its higher MGC and lower *T*_gel_ values.

Generally, triphenylmethyl protecting groups of alcohols are easily removed under acidic conditions in solution [29]. We wanted to see if this is still true for triphenylmethyl derivatives present in the gel state (e.g., gels obtained from triphenylmethyl derivatives in this study). Hence, the stability of **TPM-G12** gel (propan-1-ol) under acidic conditions was tested. Interestingly, acidification of the organogel (by adding 100 µL of 2 N HCl solution to the organogel prepared in 0.5 mL) did not bring about any visible change in its integrity. It remained stable for days without any apparent structural disintegration. If the triphenylmethyl protecting group was easily removed in the gel state, there would have been a discernible change in the structural integrity of the gel (visually at least). The control test in solution (gelator solubilised in DCM followed by addition of acid) showed that the triphenylmethyl group is easily removed as expected (as monitored by TLC). This observation clearly demonstrates that the triphenylmethyl derivative of a primary alcohol in the gel state (self-aggregated into nano- and micro-scale structures) is impervious to an acidic solution in contrast to its unstable solution-phase behavior.

### Differential scanning calorimetry

**Figure 3:**
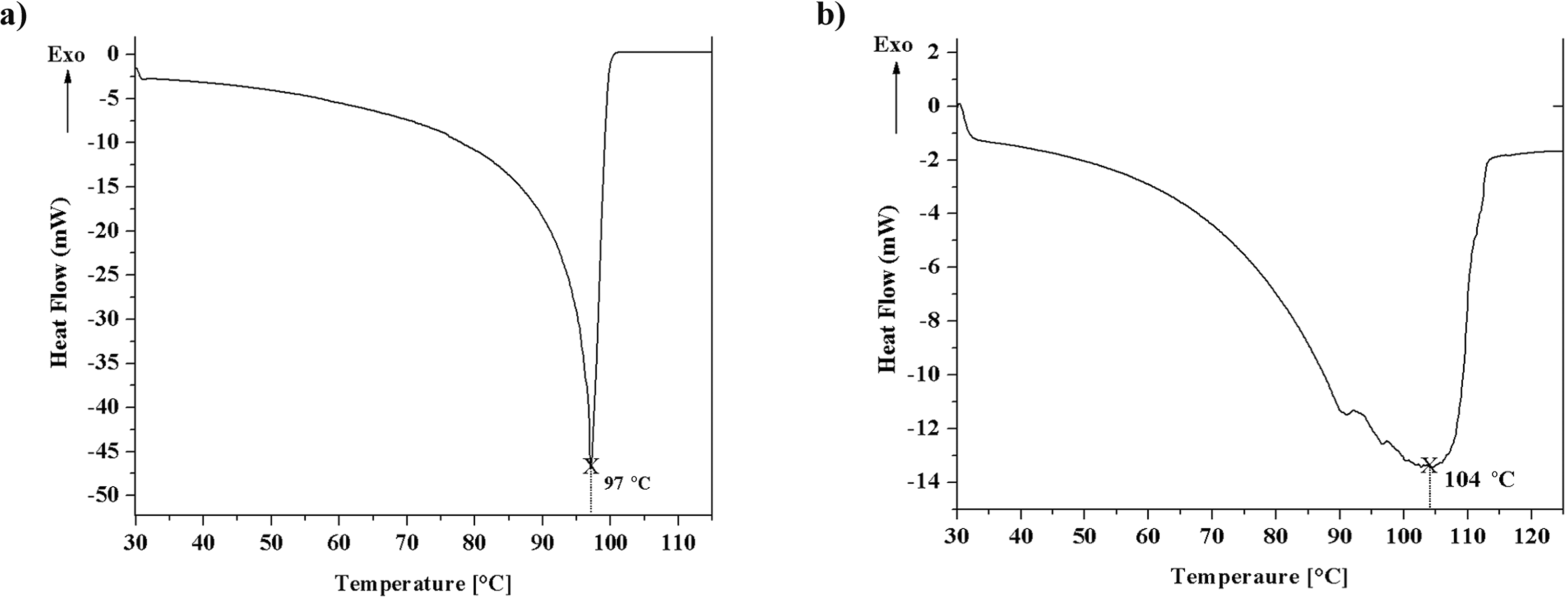
DSC thermograms of gels prepared from **TPM-G12** in (a) Propan-1-ol and (b) DMSO.

Differential scanning calorimetry (DSC) of gels prepared from **TPM-G12** (in propan-1-ol and DMSO) was carried out to study the gel-sol transition (*T*_gel_) behaviour. Figure 3 shows the DSC heating curves of **TPM-G12** prepared at 2% w/v. When a system changes from an ordered to a disordered state such as the transition from gel to sol, an endothermic peak in the DSC scan is expected which is observed here [32]. For gel prepared in propan-1-ol, the *T*_gel_ (melting of gel) is observed to be 97 °C, whereas for DMSO gel the value is 104 °C. Gel melting profile for propan-1-ol gel is much sharper compared to DMSO gel. It may also be noted here that the *T*_gel_ determined from DSC is somewhat higher in comparison to values obtained from the dropping ball method (Figure 2).

### Gel morphologies

**Figure 4:**
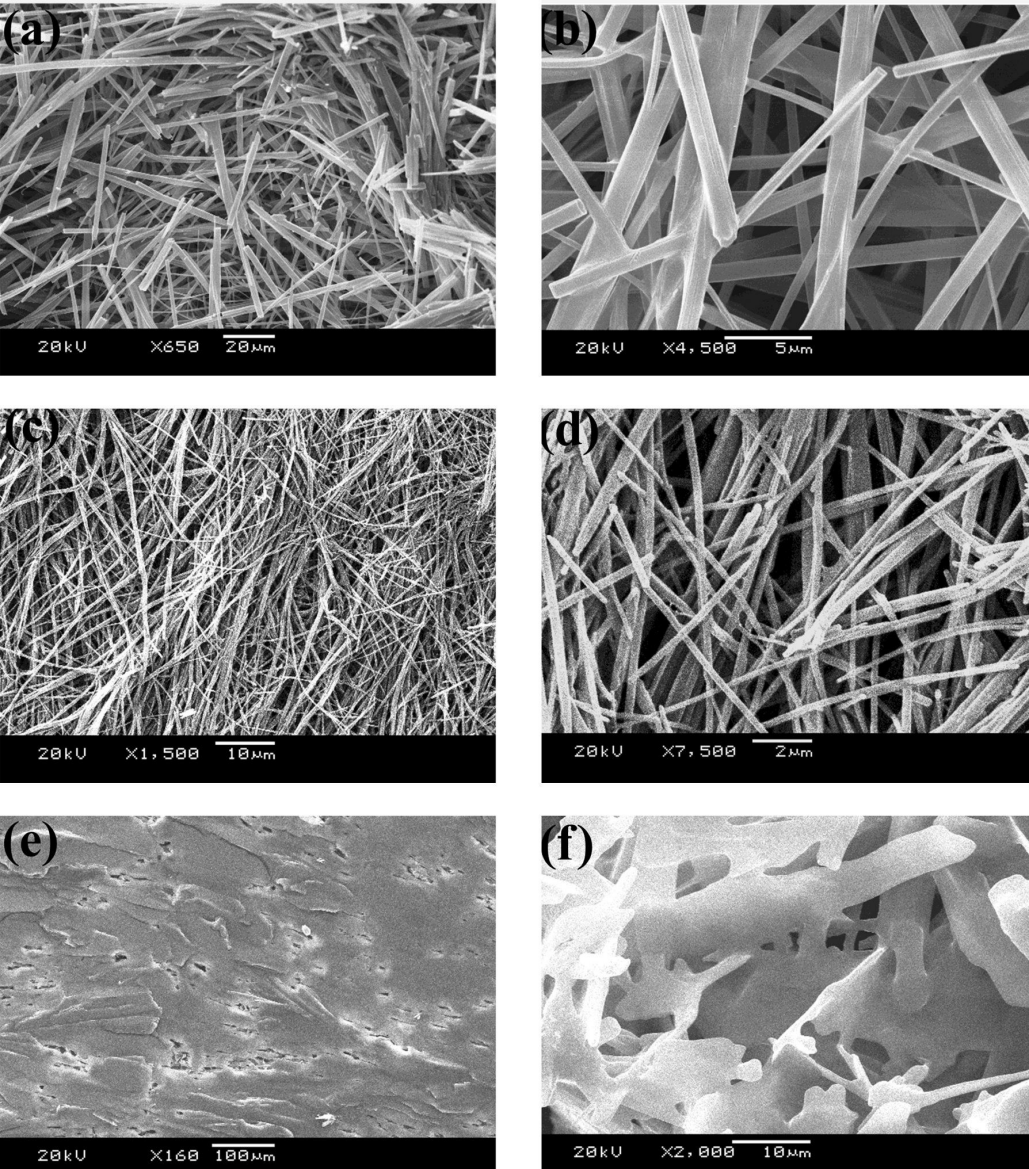
SEM images of the dried gels. **TPM-G12** in propan-1-ol (a) and (b), in DMSO (c) and (d); **TPM-G5** in DMSO (e) and (f). (a), (c), (e) are of lower magnification while (b), (d), (f) are of higher magnification.

To understand the microscopic structures and morphologies of the gels formed by the gelators, scanning electron microscopy (SEM) studies were performed with the dried gels [33]. Dried gels (xerogels) were obtained from the gels by evaporation of the solvent. The morphologies for **TPM-G12** and **TPM-G5** are drastically different (Figure 4). For **TPM-G12** (propan-1-ol and DMSO), ‘rod-like’ structures with varying sizes (in width and length) were observed. These ‘rod-like’ structures are mostly separated and not very interconnected. They are unlike highly interconnected structures which are commonly observed in fibrous networks seen for many gelators reported in the literature. The dimensions of these rods are approximately in the range of tens of nanometers to low micrometer in width and several tens of micrometers in length. Interestingly, smaller sized rod-like structures are seen in DMSO gel while larger ones are observed for propan-1-ol gel. In stark contrast, **TPM-G5** formed irregular-shaped ‘sheet-like’ structures which are highly interconnected in nature. Width and length of these sheet-like structures are in the range of several tens of micrometers.

### Rheological studies

**Figure 5:**
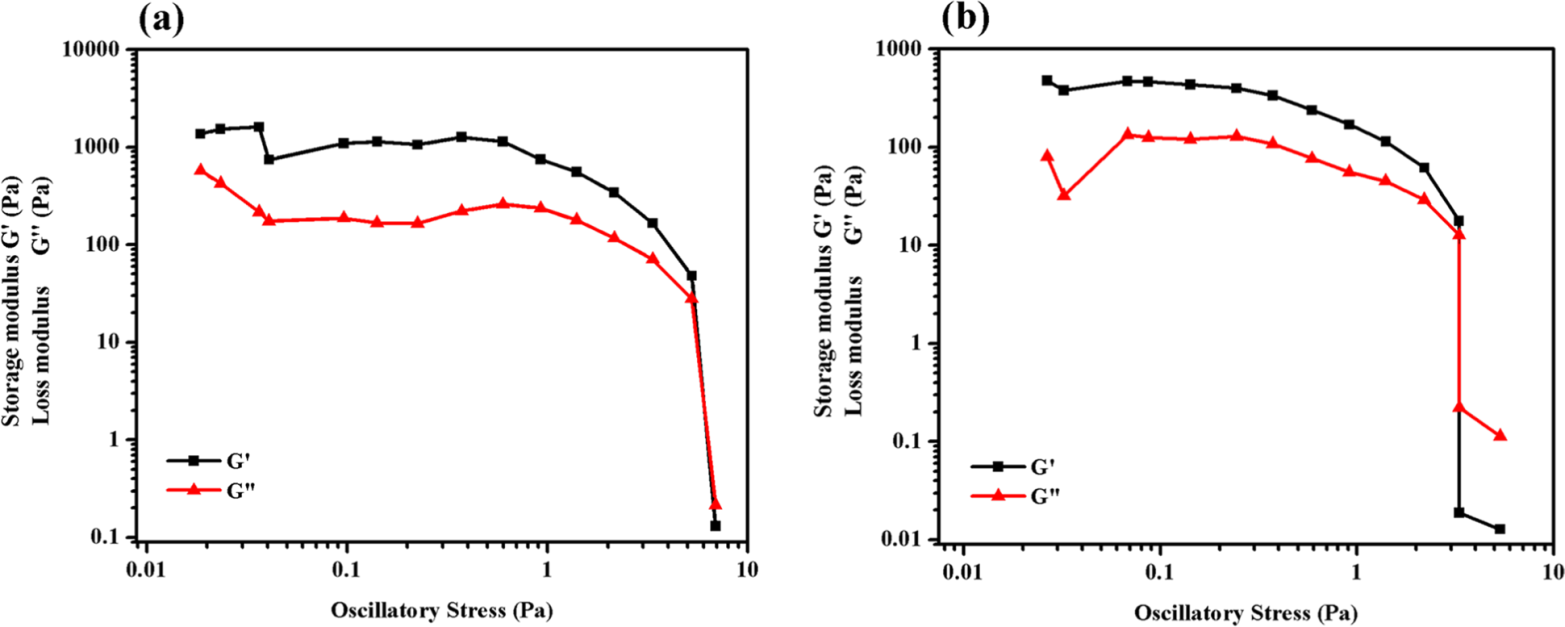
Stress sweep rheological experiment of **TPM-G12** gel (1.5% w/v) in (a) propan-1-ol (b) DMSO.

Rheological studies of **TPM-G12** gels (propan-1-ol and DMSO) were performed to understand their viscoelastic behaviour. We measured the two parameters, G‘ (storage modulus) and G“ (loss modulus) of these gels. While G‘ relates to the ability of a deformed material to store energy, G“ concerns with the flow behaviour of the material under stress [34]. In the gel state, the value of G‘ should be greater than G“ while this is reversed in the sol state. We carried out stress amplitude sweep experiment wherein G‘ and G“ were measured as a function of oscillatory shear stress at a constant oscillation frequency (Figure 5). As expected for gels, G‘ is greater than G“ for both of the organogels over a certain range of applied stress. At the low-stress region it shows a somewhat linear response, however, it shows deviation from linearity at higher stress values. Beyond a certain stress point, G‘‘ becomes higher than G‘ which is defined as the yield stress of the viscoelastic material. For these gels, similar yield stress values (≈6–7 Pa) were obtained. These values are somewhat on the lower side.

### FTIR studies

**Figure 6:**
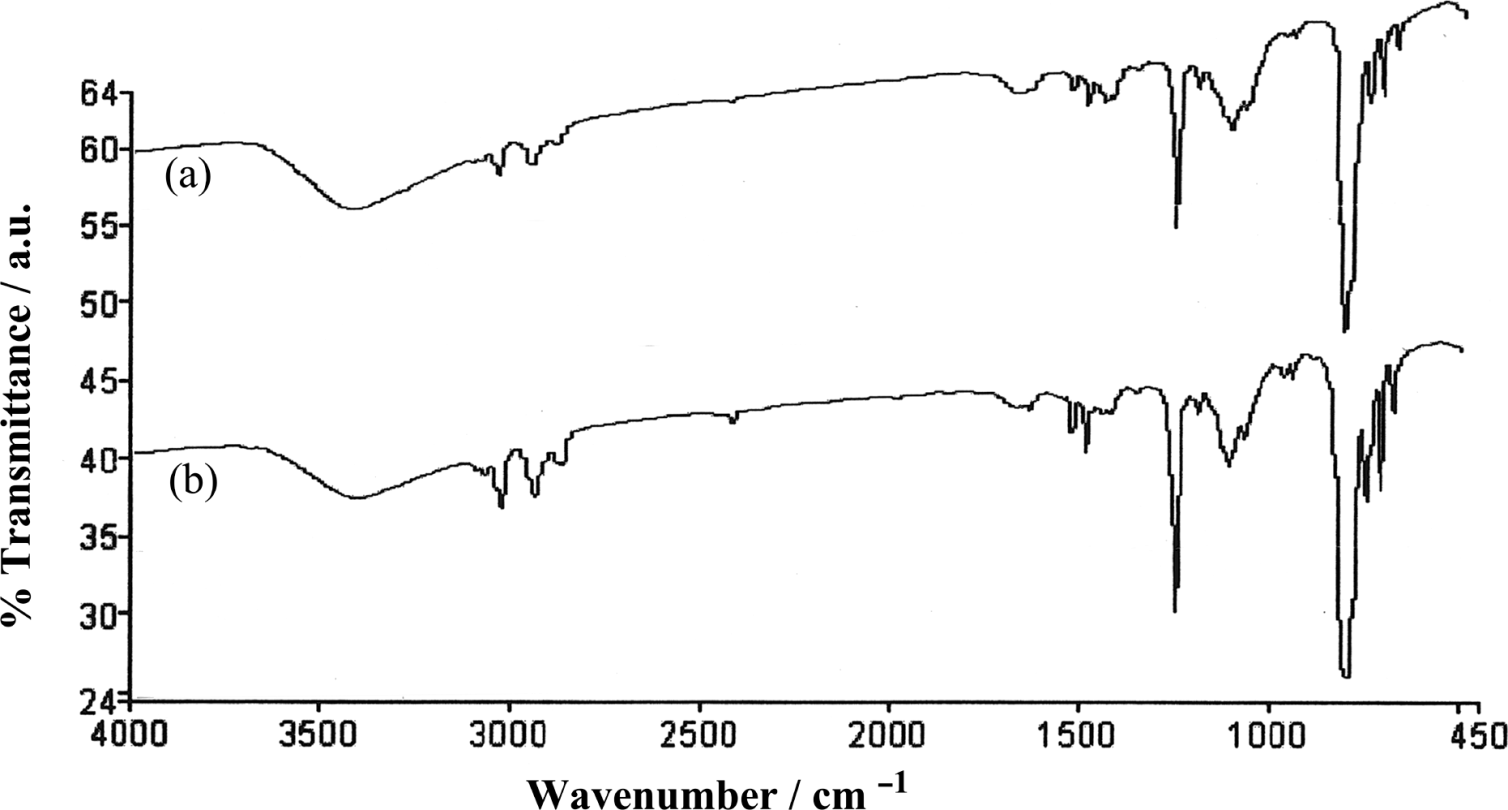
FTIR spectra of gelator **TPM-G12** in (a) CHCl_3_ and (b) xerogel in KBr.

FTIR is one of the techniques used to study the influence of non-covalent interactions in the self-assembly of gelator molecules [35]. We carried out the FTIR analysis of **TPM-G12** in solution (CHCl_3_, non-self-assembled state) and xerogel (KBr, self-assembled state) and compared their differences. Since there is no structural component capable of forming relatively strong intermolecular non-covalent interactions in **TPM-G12** (e.g., hydrogen bond, ionic bond), we did not expect much difference between the two IR spectra. Indeed, this is the case as shown in Figure 6. Aromatic ring peaks for C–H str (3019 cm^−1^), out-of-plane C–H def (768 cm^−1^) and C=C str (1402 cm^–1^, 1448 cm^–1^ and 1490 cm^–1^) are seen in the solution state. Except for out-of-plane C–H def (759 cm^–1^), almost identical peak values are observed in the xerogel too. The peak value for C–O str (1216 cm^–1^) also remains the same in both the states. So, FTIR studies strongly suggest that there is an absence of strong intermolecular non-covalent interactions in the gel state.

### XRD studies and molecular packing

**Figure 7:**
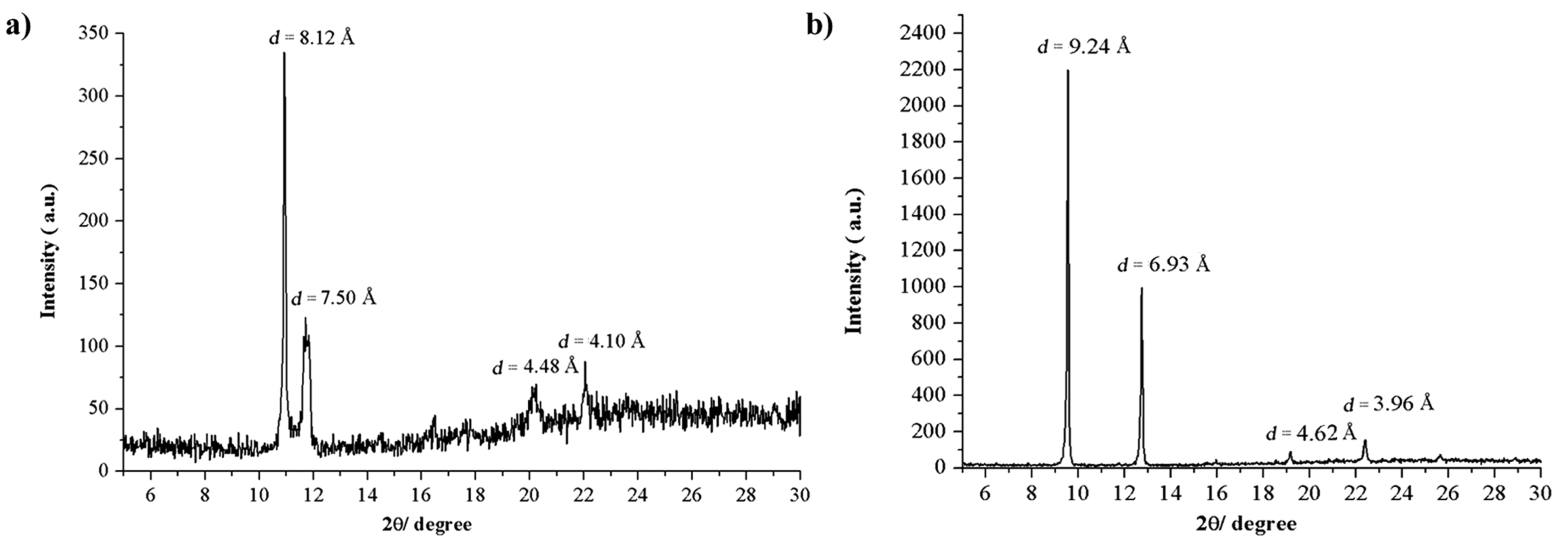
Powder X-ray diffraction patterns of xerogel of (a) **TPM-G12** from propan-1-ol and (b) **TPM-G5** from DMSO.

**Figure 8:**
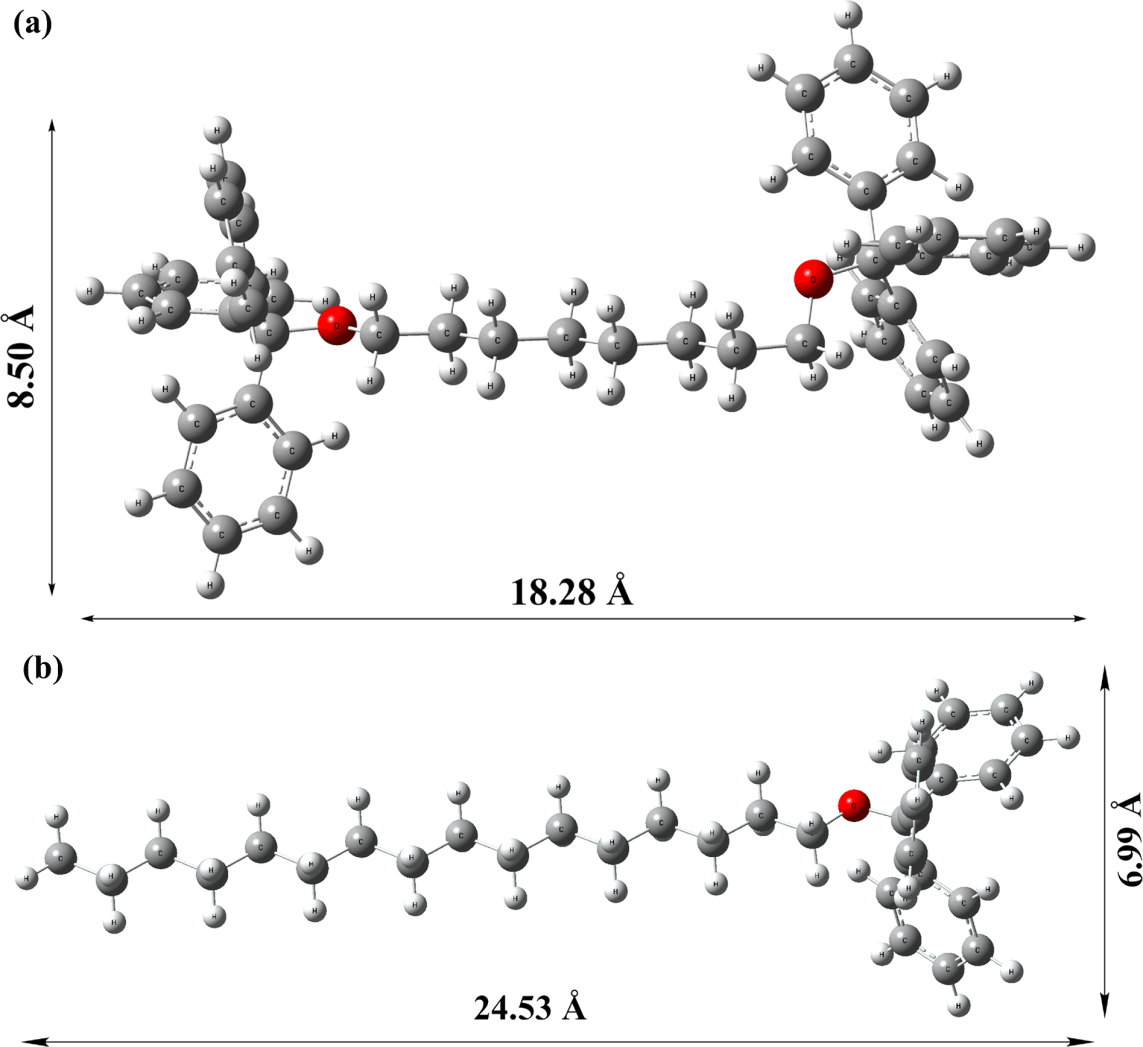
Energy minimized conformational structure of (a) **TPM-G12** and (b) **TPM-G5** obtained using B3LYP/6-31G (d, p) level of computations.

**Figure 9:**
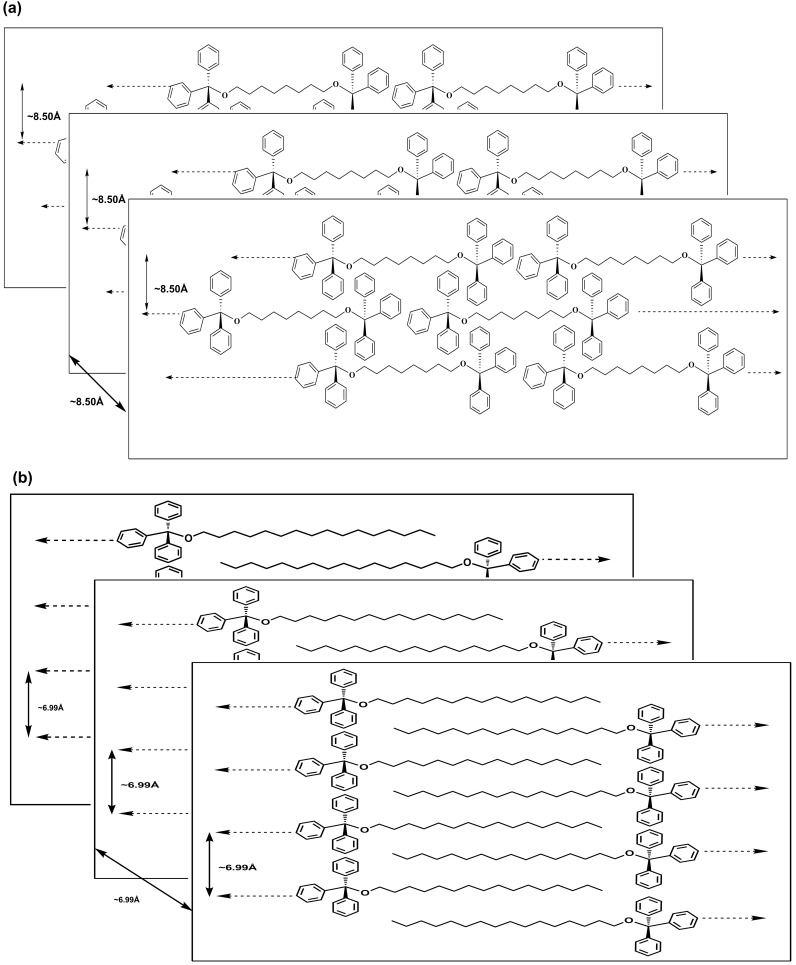
Possible molecular packing arrangement in the self-assembled gel state of (a) **TPM-G12** and (b) **TPM-G5**.

We have carried out powder XRD analysis using dried gel of **TPM-G12** (propan-1-ol) and **TPM-G5** (DMSO) to get an understanding of the molecular packing in the self-assembled gel state [36]. For **TPM-G12**, one high-intensity peak at 10.94° (2θ value), one medium-intensity peak at 11.86°, and two low-intensity peaks at 20.15° and 22.02° were observed. The corresponding d-spacing values are 8.12 Å, 7.50 Å, 4.48 Å and 4.10 Å, respectively (Figure 7a). In the energy-minimized state, **TPM-G12** adopts a symmetrical ‘dumbbell-shaped‘ structure (Figure 8a). Based on the observed d-spacing values and energy minimized molecular size, we propose a possible molecular packing arrangement of **TPM-G12** in the self-assembled gel state (Figure 9a). Self-assembly in the gel state will be driven predominantly by hydrophobic interactions between the octyl alkyl chains and triphenylmethyl groups. The gelator molecules may be arranged linearly in a head-to-tail (end-to-end) direction. These linear arrays can then interact laterally with two triphenylmethyl groups in close proximity to neighbouring octyl chains resulting in a roughly two-dimensional planar packing arrangement. The distance between any two adjacent linear arrays will be approximately equal to (or less than) the width of the **TPM-G12** molecule (8.50 Å). Subsequently, different layers of these two-dimensional planar structures can aggregate together to give the three-dimensional microstructure of the gel. The distance between any two planar two-dimensional structures will be similar to the width of **TPM-G12** (8.50 Å). The observed d-spacing value of 8.12 Å from powder XRD experiment correlates closely with this value. The weak intensity peak at 22.02° (4.10 Å) may result from π–π stacking between benzene rings of adjacent triphenylmethyl groups which are likely to be involved in self-assembly of the gelator molecules.

For **TPM-G5**, two high-intensity peaks at 9.56° (2θ value) and 12.75°, and two low-intensity peaks at 19.17° and 22.39° were observed (Figure 7b). The d-spacing values are 9.24 Å, 6.93 Å, 4.62 Å and 3.96 Å, respectively. Figure 8b shows the energy minimized conformational structure of **TPM-G5**. Based on the observed d-spacing values and energy minimized molecular conformation of **TPM-G5**, a possible molecular packing arrangement in the self-assembled gel state is presented (Figure 9b). One possible arrangement is an interdigitated bilayer structure. The molecules are oriented side by side such that each hexadecyl alkyl chain is sandwiched between two such chains. The high-intensity peak at 12.75° (d-spacing value of 6.93 Å which is comparable to the width of **TPM-G5**, 6.99 Å) can be attributed to the distance between adjacent linear molecular arrays and also between two-dimensional planar arrays. The weak intensity peak at 22.39° (3.96 Å) can be attributed to the presence of π–π stacking between benzene rings of adjacent triphenylmethyl groups.

### Dye absorption studies

**Figure 10:**
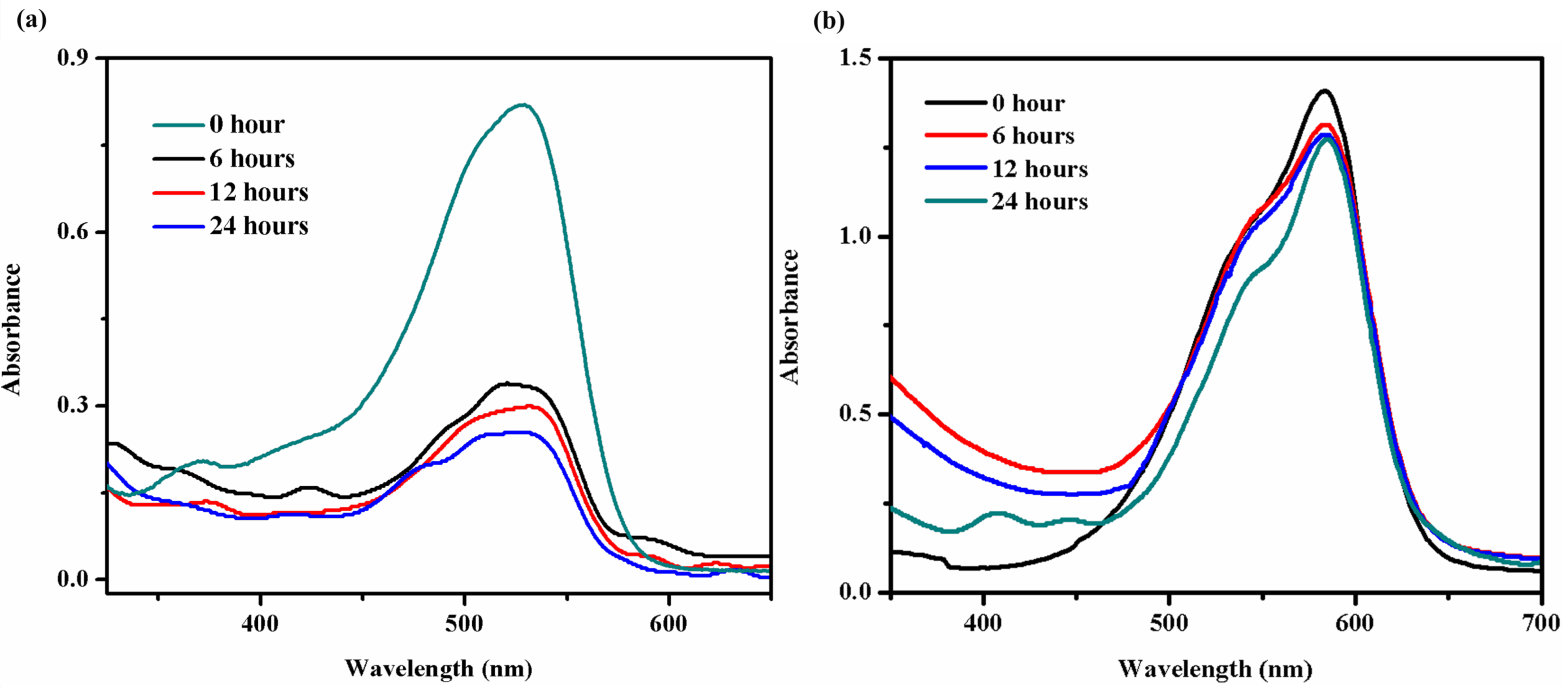
Time-dependent UV–vis absorption profile of (a) Direct Red 80 (b) Crystal Violet aqueous dye solution (0.02 mM) by **TPM-G12** gel (1% w/v in propan-1-ol).

Dyes are commercially important and are widely used in many industries. But most of these dyes are toxic and harmful to the environment. Discharge of waste water generated during use of these water-soluble dyes without proper pretreatment creates a serious environmental and health hazard. Hence, development of simple and reliable methods to remove dyes from the industrial waste water is becoming increasingly important. Conventionally, this has been achieved through the use of materials like clay, porous silica, polymers, charcoals, etc. [37]. More recently, some gels derived from low molecular weight compounds have been shown to possess the ability to absorb dyes [38–39]. Hence, small molecules based molecular gels have recently emerged as a new class of materials which can be used in the removal of water-soluble toxic dyes. We reasoned that **TPM-G12** gel (being non-polar) can perhaps be utilized for removal of water-soluble dyes having appreciable hydrophobic character (perhaps with a large π surface area, e.g., Direct Red 80). The hypothesis being hydrophobic interactions between the non-polar self-assembled **TPM-G12** microstructures with Direct Red 80 may facilitate dye absorption and removal. Figure 10a shows the time-dependent dye absorption profile of Direct Red 80 by **TPM-G12** gel (propan-1-ol) as measured by UV–vis technique. The dye absorption efficiency is 58, 63 and 69% after 6, 12, and 24 h of incubation, respectively. So, **TPM-G12** gel showed a fairly good absorption capacity for Direct Red 80. In contrast to this observation, the same gel showed very low absorption (9% after 24 h) of crystal violet dye (much polar compared to Direct Red 80) which reinforces our hypothesis that hydrophobic interactions between the gel microstructures and dye molecules play an important role in the absorption process (Figure 10b).

## Conclusion

We have developed a new class of organogelators based on triphenylmethyl derivatives of simple and easily available primary alcohols. One big advantage of these gelators is their straightforward synthesis and easy accessibility. Overall, gelation efficiency of some of the triphenylmethyl derivatives was moderate to excellent with minimum gelation concentration in the range of 0.5–4.0%, w/v. 1,8-Bis(trityloxy)octane, the ditrityl derivative of 1,8-octanediol was found to be an excellent gelator of some polar solvents. Detailed characterizations of the gel were carried out using scanning electron microscopy, FTIR spectroscopy, rheology and powder XRD techniques. Based on its absorption profile for a water soluble dye, this gel can potentially be used as a dye removal agent from waste water. The results strongly suggest that hydrophobic interactions alone can mediate gelation of polar solvents and this approach can be exploited in the design of new gelators. We are further pursuing this line of investigation by exploring for hitherto undiscovered hydrophobic structural components which can be incorporated in the design of new efficient gelators.

## Supporting Information

File 1 Experimental part.
