# Supplementary material for: A new class of organogelators based on triphenylmethyl derivatives of primary alcohols: hydrophobic interactions alone can mediate gelation
Source: Beilstein J Org Chem. 2017 Jan 23;13:138–49. doi: 10.3762/bjoc.13.17 (PMC5301947; doi:10.3762/bjoc.13.17)
Supplement: File 1 — Experimental part. [file Beilstein_J_Org_Chem-13-138-s001.pdf]

Supporting Information

for

A new class of organogelators based on triphenylmethyl derivatives of primary alcohols: hydrophobic interactions alone can mediate gelation

Wangkhem P. Singh and Rajkumar S. Singh*

Address: Organic Materials Research Laboratory, Department of Basic Sciences & Social Sciences, North-Eastern Hill University, Shillong-793022, Meghalaya, India

Email: Rajkumar S. Singh* - rksunil3000@yahoo.co.in

*Corresponding author

Experimental part

Contents:

1. Materials and instruments
2. Synthesis
3. Organogel preparation
4. Minimum gelation concentration (MGC) determination
5. Gel–sol transition (T_{gel}) temperature measurement
6. Differential scanning calorimetry
7. SEM analysis
8. Rheology
9. FTIR analysis
10. XRD procedure
11. Dye absorption studies
12. Geometrical optimization method

1. Materials and instruments

1-Octanol, 1-decanol, 1-dodecanol, 1-tetradecanol, 1-hexadecanol, diethylene glycol, triethylene glycol, silica gel (60-120 mesh), all solvents for gelation studies, petroleum ether (60-80), and ethyl acetate were procured from SD Fine Chemicals, India. 1,6-Hexanediol, 1,8-octanediol, 1,10-decanediol, trityl chloride, TLC silica gel 60-F_{254} plates were purchased from Sigma-Aldrich, India. ^{1}H NMR, ^{13}C NMR were recorded using 400 MHz FT-NMR spectrometer (Bruker Avance II 400 MHz) at SAIF-NEHU, Shillong and 300 MHz FT-NMR Spectrometer (Bruker Avance DPX-300) at SAIF, CSIR-CDRI, Lucknow. MALDI–TOFMS and ESI–TOFMS were done with ABSciex TOF/TOF Series Explorer 7000 & Synapt G2 HDMS (Waters) respectively at the Advanced Instrumentation Research facility-JNU, New Delhi and with Bruker Ultraflex TOF/TOF at RGCB, Thiruvananthapuram. FT-IR was performed with Perkin Elmer Spectrum II spectrometer at SAIF, CSIR-CDRI, Lucknow. SEM analysis was carried out using Jeol JSM-6360 at SAIF-NEHU, Shillong. XRD analysis was carried out using XPert Pro (PANalytical) at SAIF-Gauhati University, Guwahati. Differential Scanning Calorimetry was performed using Mettler Toledo DSC 822 at STIC-Cochin University. Rheology studies were carried out on an Anton Parr MCR302 rheometer at the Indian Institute of Technology, Indore. A Hitachi U-3900 UV–vis spectrophotometer was used for dye absorption studies.

2. Synthesis

2.1 General synthetic procedure for Series 1 compounds (TPM-G1 - TPM-G5)

Synthetic procedure for **TPM-G1** is given below as a representative example. The same procedure was followed for the synthesis of the remaining compounds (**TPM-G2 – TPM-G5**) using similar molar scales. Trityl chloride (641 mg, 2.30 mmol) dissolved in 4 mL of dichloromethane (DCM) was added to a stirred solution of octan-1-ol (300 mg, 2.30 mmol) in DCM at room temperature in a 50 mL round bottom flask under nitrogen atmosphere. Triethylamine (232 mg, 2.30 mmol) diluted in 2 mL of DCM was added dropwise to the reaction mixture and stirring was continued for 24 h at room temperature. The reaction mixture was taken in ≈30 mL DCM and washed three times with water. The organic extract was dried over

anhydrous sodium sulfate and concentrated by rotary evaporation. The crude product was purified by column chromatography over silica gel 60–120 mesh using petroleum ether/ethyl acetate (99:1) as the eluent to give compound **TPM-G1**.

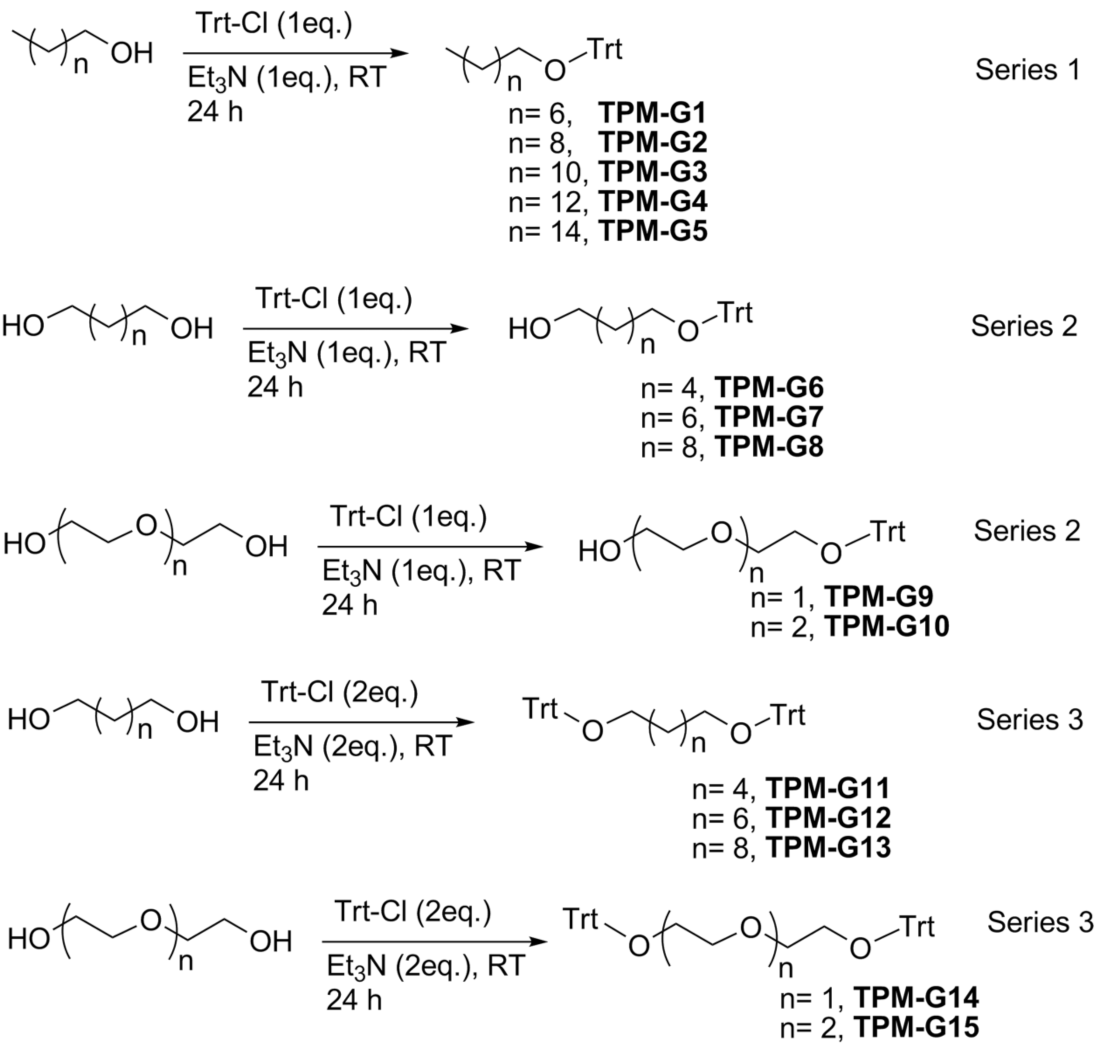

Figure S1: Synthetic routes of **TPM-G1–TPM-G15**.

(Octyloxymethanetriyl)tribenzene (**TPM-G1**). Semisolid (780 mg, yield 91 %). ^{1}H NMR ($CDCl_3$, 400 MHz,): δ (ppm) 0.86- 0.88 (t, 3H, J = 6.7 Hz), 1.24-1.48 (m, 10H), 1.57-1.64 (m, 2H), 3.01-3.05 (t, 2H, J = 6.7 Hz), 7.19- 7.23 (t, 3H, J = 7.3 Hz), 7.26-7.30 (t, 6H, J = 7.3 Hz), 7.44-7.46 (d, 6H, J = 7.3 Hz). ^{13}C NMR ($CDCl_3$, 100 MHz): δ (ppm) 144.80, 128.94, 127.88, 127.00, 86.50, 63.92, 32.06, 30.28, 29.70, 29.47, 26.50, 22.88, 14.32.

(Decyloxymethanetriyl)tribenzene (**TPM-G2**). Semisolid (Yield 64%). ^{1}H NMR ($CDCl_3$, 400 MHz,): δ (ppm) 1.03- 1.06 (t, 3H, *J* = 6.7 Hz), 1.42 - 1.52 (m, 14H), 1.77 - 1.80 (m, 2H), 3.21-3.24 (t, 2H, *J* = 6.1Hz), 7.33- 7.36 (t, 3H, *J* = 7.3Hz), 7.40-7.44 (t, 6H, *J* = 7.3Hz), 7.61-7.63 (d, 6H, *J* = 7.3 Hz). ^{13}C NMR ($CDCl_3$, 100 MHz): δ (ppm) 144.80, 128.94, 127.88, 127.00, 86.50, 63.92, 32.13, 30.29, 29.81, 29.80, 29.74, 29.55, 26.50, 22.90, 14.33. ESI-TOF (MS): *m/z* calcd for $C_{29}H_{36}ONa$: 423.277 $[M+Na]^+$; found 423.164.

(Dodecyloxymethanetriyl)tribenzene (**TPM-G3**). White solid (Yield 60%), mp: 24-25 ^{o}C. ^{1}H NMR ($CDCl_3$, 400 MHz,): δ (ppm) 0.78- 0.81 (t, 3H, *J* = 7.3 Hz), 1.17 - 1.43 (m, 18H), 1.50 - 1.57 (q, 2H), 2.94-2.97 (t, 2H, *J* = 6.1 Hz), 7.11- 7.15 (t, 3H, *J* = 7.3 Hz), 7.18-7.22 (t, 6H, *J* = 7.3 Hz), 7.35-7.37 (d, 6H, *J* = 7.3 Hz). ^{13}C NMR ($CDCl_3$, 75.5 MHz): δ (ppm) 144.81, 128.95, 127.88, 126.99, 86.51, 63.93, 32.15, 30.30, 29.87, 29.75, 29.58, 26.51, 22.91, 14.33.

(Tetradecyloxymethanetriyl)tribenzene (**TPM-G4**). White solid (Yield 76%), mp: 38-39 ^{o}C. ^{1}H NMR ($CDCl_3$, 400 MHz,): δ (ppm) 0.78- 0.81 (t, 3H, *J* = 6.7 Hz), 1.17 - 1.42 (m, 22H), 1.50 - 1.55 (q, 2H), 2.94-2.97 (t, 2H, *J* = 6.7 Hz), 7.11- 7.15 (t, 3H, *J* = 7.3 Hz), 7.18-7.22 (t, 6H, *J* = 7.3 Hz), 7.35-7.37 (d, 6H, *J* = 7.3 Hz). ^{13}C NMR ($CDCl_3$, 100 MHz): δ (ppm) 143.49, 127.65, 126.63, 125.73, 85.20, 62.64, 30.90, 29.03, 28.67, 28.66, 28.64, 28.61, 28.60, 28.57, 28.50, 28.34, 25.23, 21.67, 13.11. Elemental analysis cald (%) for $C_{33}H_{44}O$: C 86.76, H 9.72; found: C 86.88, H 9.75.

(Hexadecyloxymethanetriyl)tribenzene (**TPM-G5**). White solid (Yield 76%), mp: 44-45 ^{o}C. ^{1}H NMR ($CDCl_3$, 400 MHz,): δ (ppm) 0.78- 0.81 (t, 3H, *J* = 6.7 Hz), 1.17 - 1.45 (m, 26H), 1.50 - 1.55 (q, 2H), 2.94-2.97 (t, 2H, *J* = 6.7 Hz), 7.12- 7.15 (t, 3H, *J* = 7.3 Hz), 7.19-7.22 (t, 6H, *J* = 7.3 Hz), 7.36-7.37 (d, 6H, *J* = 7.3 Hz). ^{13}C NMR ($CDCl_3$, 100 MHz): δ (ppm) 144.81, 128.95, 127.89, 127.00, 86.51, 63.93, 32.16, 30.30, 29.93, 29.94, 29.91, 29.90, 29.88, 29.87, 29.86, 29.83, 29.76, 29.60, 26.51, 22.93, 14.35. ESI-TOF (MS): *m/z* calcd for $C_{35}H_{48}ONa$: 507.384 $[M+Na]^+$; found 507.170.

2.2 General synthetic procedure for Series 2 compounds (TPM-G6 - TPM-G10)

Synthetic procedure for **TPM-G6** is described below as a representative example. The same procedure was used for the synthesis of the remaining compounds (TPM-G7 – TPM-G10) at similar molar scales. Trityl chloride (697 mg, 2.50 mmol)

dissolved in 4 mL of DCM was added to a stirred solution of 1,6-hexanediol (300 mg, 2.50 mmol) in DCM at room temperature in a 50 mL round bottom flask under nitrogen atmosphere. Triethylamine (253 mg, 2.50 mmol) diluted in 2 mL of DCM was added dropwise to the reaction mixture and stirring continued for 24 h at room temperature. The reaction mixture was taken in ~30 mL DCM and washed three times with water. The organic extract was dried over anhydrous sodium sulfate and concentrated by rotary evaporation. The crude product was purified by column chromatography over silica gel 60-120 mesh using petroleum ether/ethyl acetate (9:1) as the eluent to give compound TPM-G6.

6-(trityloxy)hexan-1-ol (**TPM-G6**). White solid (370 mg, yield 41%), mp: 68-69 ^{o}C. ^{1}H NMR ($CDCl_3$, 400 MHz,): δ (ppm) 1.28-1.66 (m, 8H), 3.00-3.07 (m, 2H), 3.59-3.66 (m, 2H), 7.18- 7.30 (m, 9H), 7.41-7.45 (m, 6H). ^{13}C NMR ($CDCl_3$, 75.5 MHz): δ (ppm) 144.72, 128.90, 127.88, 127.43, 86.53, 63.76, 63.14, 32.92, 30.20, 26.34, 25.80. ESI-TOF (MS): *m/z* calcd for $C_{25}H_{28}O_2Na$: 383.213 $[M+Na]^+$; found 383.123.

8-(trityloxy)octan-1-ol (**TPM-G7**). Semisolid (Yield 60%). ^{1}H NMR ($CDCl_3$, 400 MHz,): δ (ppm) 1.22-1.57 (m, 12H), 2.94-2.97 (t, 2H, *J* = 6.7 Hz), 3.53-3.57 (t, 2H, *J* = 6.7 Hz), 7.13- 7.16 (t, 3H, *J* = 7.3 Hz), 7.20-7.23 (t, 6H, *J* = 7.3 Hz), 7.36-7.38 (d, 6H, *J* = 7.3 Hz). ^{13}C NMR ($CDCl_3$, 75.5 MHz): δ (ppm) 144.76, 128.91, 127.87, 126.98, 86.50, 63.85, 63.23, 32.96, 30.23, 29.89, 29.63, 26.40, 25.88. ESI-TOF (MS): *m/z* calcd for $C_{27}H_{32}O_2Na$: 411.245 $[M+Na]^+$; found 411.152.

10-(trityloxy)decan-1-ol (**TPM-G8**). White solid (Yield 32%), mp: 31-32 ^{o}C. ^{1}H NMR ($CDCl_3$, 400 MHz,): δ (ppm) 1.18-1.57 (m, 16H), 2.94-2.97 (t, 2H, *J* = 7.3 Hz), 3.53-3.57 (t, 2H, *J* = 6.7 Hz), 7.13- 7.16 (t, 3H, *J* = 7.4 Hz), 7.20-7.23 (t, 6H, *J* = 7.3 Hz), 7.36-7.38 (d, 6H, *J* = 7.3 Hz). ^{13}C NMR ($CDCl_3$, 100 MHz): δ (ppm) 144.77, 128.92, 127.87, 126.98, 86.49, 63.89, 63.26, 33.00, 30.26, 29.75, 29.70, 29.69, 29.61, 26.47, 25.94. ESI-TOF (MS): *m/z* calcd for $C_{29}H_{36}O_2Na$: 439.277 $[M+Na]^+$; found 439.181.

2-(2-(trityloxy)ethoxy)ethanol (**TPM-G9**). White solid (Yield 30%), mp: 110-113 ^{o}C. ^{1}H NMR ($CDCl_3$, 400 MHz,): δ (ppm) 3.17- 3.20 (t, 2H, *J* = 5.2 Hz), 3.53-3.55 (t, 2H, *J* = 4.4 Hz), 3.59-3.61 (t, 2H, *J* = 5.2 Hz), 3.53-3.55 (t, 2H, *J* = 4.4 Hz), 7.14- 7.17 (t, 3H, *J* = 7.2 Hz), 7.19-7.22 (t, 6H, *J* = 7.6 Hz), 7.37-7.39 (d, 6H, *J* = 7.6 Hz). ^{13}C NMR ($CDCl_3$, 75.5 MHz): δ (ppm) 144.39, 128.91, 127.99, 127.20, 86.92, 72.50, 71.01,

63.73, 62.07. MALDI-TOF (MS): *m/z* calcd for $C_{23}H_{24}O_3Na$: 371.408 $[M+Na]^+$; found 371.191.

2-(2-(2-(trityloxy)ethoxy)ethoxy)ethanol (**TPM-G10**). Semisolid (Yield 42%). ^{1}H NMR ($CDCl_3$, 400 MHz,): δ (ppm) 3.24- 3.26 (t, 2H, *J* = 4.9 Hz), 3.61-3.63 (t, 2H, *J* = 4.9 Hz), 3.67-3.73 (m, 8H), 7.27-7.34 (m, 9H), 7.45-7.47 (d, 6H, *J* = 7.3 Hz). ^{13}C NMR ($CDCl_3$, 100 MHz): δ (ppm) 147.11, 128.90, 127.95, 127.15, 86.82, 72.72, 71.01, 70.90, 70.72, 63.49, 61.99. MALDI-TOF (MS): *m/z* calcd for $C_{25}H_{28}O_4Na$: 415.459 $[M+Na]^+$; found 415.420.

2.3 General synthetic procedure for Series 3 compounds (TPM-G11 - TPM-G15)

Synthetic procedure for **TPM-G11** is described below as a representative example. The same procedure was used for the synthesis of the remaining compounds (TPM-G12 – TPM-G15) at similar molar scales. Trityl chloride (1.40 g, 5.0 mmol) dissolved in 6 mL of DCM was added to stirred solution of 1,6-hexanediol (300 mg, 2.50 mmol) in DCM at room temperature in a 50 mL round bottom flask under nitrogen atmosphere. Triethylamine (505 mg, 5.0 mmol) diluted in 2 mL of DCM was added dropwise to the reaction mixture and stirring continued for 24 h at room temperature. The reaction mixture was taken in ~30 mL DCM and washed three times with water. The organic extract was dried over anhydrous sodium sulfate and concentrated by rotary evaporation. The crude product was purified by column chromatography over silica gel 60-120 mesh using petroleum ether/ethyl acetate (98:2) as the eluent to give compound TPM-G11.

1,6-Bis(trityloxy)hexane (**TPM-G11**). White solid (270 mg, yield 18%), mp: 131-132 ^{o}C. ^{1}H NMR ($CDCl_3$, 400 MHz,): δ (ppm) 1.28-1.33 (q, 4H), 1.52-1.61 (m, 4H), 3.00-3.03 (t, 4H, *J* = 6.8 Hz), 7.19- 7.22 (t, 6H, *J* = 7.6 Hz), 7.24-7.27 (t, 12H, *J* = 7.6 Hz), 7.41-7.43 (d, 12H, *J* = 7.6 Hz). ^{13}C NMR ($CDCl_3$, 75.5 MHz): δ (ppm) 144.75, 128.92, 128.14, 126.99, 86.50, 63.79, 30.22, 26.36. MALDI-TOF (MS): *m/z* calcd for $C_{44}H_{42}O_2Na$: 625.763 $[M+Na]^+$; found 625.967.

1,8-Bis(trityloxy)octane (**TPM-G12**). White solid (Yield 26%), mp: 125-126 ^{o}C. ^{1}H NMR ($CDCl_3$, 400 MHz,): δ (ppm) 1.22-1.61 (m, 12H), 3.00-3.03 (t, 4H, *J* = 6.7 Hz), 7.19- 7.23 (t, 6H, *J* = 7.3 Hz), 7.26-7.30 (t, 12H, *J* = 8.5 Hz), 7.43-7.45 (d, 12H, *J* =

8.5 Hz). ^{13}C NMR ($CDCl_3$, 100 MHz): δ (ppm) 144.75, 128.92, 127.88, 126.99, 86.49, 63.86, 30.25, 29.64, 26.43. MALDI-TOF (MS): *m/z* calcd for $C_{46}H_{46}O_2Na$: 653.815 $[M+Na]^+$; found 653.343.

1,10-Bis(trityloxy)decane (**TPM-G13**). Semisolid (Yield 44%). ^{1}H NMR ($CDCl_3$, 400 MHz,): δ (ppm) 1.22-1.62 (m, 16H), 3.01-3.04 (t, 4H, *J* = 6.7 Hz), 7.19- 7.23 (t, 6H, *J* = 7.3 Hz), 7.26-7.30 (t, 12H, *J* = 7.3 Hz), 7.43-7.45 (d, 12H, *J* = 7.3 Hz). ^{13}C NMR ($CDCl_3$, 100 MHz): δ (ppm) 144.55, 128.72, 127.70, 126.81, 86.27, 63.70, 30.07, 29.56, 29.54, 26.29. MALDI-TOF (MS): *m/z* calcd for $C_{48}H_{50}O_2Na$: 681.867 $[M+Na]^+$; found 681.395.

1,1,1,9,9,9-Hexaphenyl-2,5,8-trioxanonane (**TPM-G14**). White solid (Yield 25%), mp: 145-147 ^{o}C. ^{1}H NMR ($CDCl_3$, 400 MHz,): δ (ppm) 3.24-3.26 (t, 4H, *J* = 5.2 Hz), 3.69-3.71 (t, 4H, *J* = 5.2 Hz), 7.18- 7.30 (m, 18H), 7.46-7.49 (m, 12H). ^{13}C NMR ($CDCl_3$, 100 MHz): δ (ppm) 144.40, 128.97, 127.98, 127.09, 86.70, 71.04, 63.74. ESI-TOF (MS): *m/z* calcd for $C_{42}H_{38}O_3Na$: 613.293 $[M+Na]^+$; found 613.172.

1,1,1,12,12,12-Hexaphenyl-2,5,8,11-tetraoxadodecane (**TPM-G15**). White solid (Yield 17%), mp: 150-152 ^{o}C. ^{1}H NMR ($CDCl_3$, 400 MHz,): δ (ppm) 3.22-3.25 (t, 4H, *J* = 5.2 Hz), 3.69-3.71 (m, 8H), 7.18- 7.28 (m, 18H), 7.44-7.46 (d, 12H, *J* = 7.3 Hz). ^{13}C NMR ($CDCl_3$, 100 MHz): δ (ppm) 144.36, 128.94, 127.96, 127.11, 86.77, 71.11, 70.98, 63.58. ESI-TOF (MS): *m/z* calcd for $C_{44}H_{42}O_4Na$: 657.325 $[M+Na]^+$; found 657.197.

3. Organogel preparation

The required amount of compound was taken in 0.5 mL of solvent in a screw-capped glass vial. It was heated slowly on a hot plate till the solid dissolved completely and left to cool to room temperature and observation noted down after 0.5–2 h. Stable gel formation was confirmed by inversion of the glass vial.

4. Minimum gelation concentration (MGC) determination

Different concentrations of the compounds (% w/v) were taken in the required solvent and gelation test performed as described above. MGC is defined as the minimum concentration at which a stable gel is formed.

5. Gel–sol transition (T_{gel}) temperature measurement

T_{gel} values were determined using the dropping ball method. A glass bead (weighing 742 mg, 3.96 mm in diameter) was placed at the top of the gel in a glass vial. This glass vial was placed in an oil bath and the temperature was raised slowly at a rate of 3 ^{o}C per minute. T_{gel} is defined as the temperature at which the glass bead reached the bottom of the glass vial.

6. Differential scanning calorimetry

Gels prepared in different solvents (propan-1-ol and DMSO) were taken in DSC ampules and measurement taken in the range of 30–125 $^{\circ}$C at a heating rate of 5 °C/min.

7. SEM analysis

A small volume of a hot solution of the gelator was placed on a glass coverslip and cooled to room temperature to allow gel formation. The gel was kept at ambient temperature overnight and then vacuum dried. The glass cover slip with the dried gel (xerogel) was transferred on a cylindrical brass stub and coated with Au. SEM images were obtained with an accelerating voltage of 20 kV.

8. Rheology

Rheology study was carried out on an Anton Parr MCR 302 instrument having cone and plate geometry. Gel (1.5 % w/v in propan-1-ol and DMSO) was carefully placed on the plate so that there was no air gap with the cone. Stress amplitude sweep experiments were carried out (in the range of 0.01-500 Pa) at a constant oscillation frequency of 1Hz at 25 ^{o}C. G’ and G” were then measured as a function of oscillatory shear stress.

9. FTIR analysis

FTIR spectra of the gelator **TPM-G12** in solution ($CHCl_3$) and dried gel (KBr pellet) were recorded with Perkin Elmer Spectrum II spectrometer.

10. XRD analysis

A few drops of the hot gelator solution was poured on a glass slide and cooled to room temperature till a gel was formed. It was vacuum dried to prepare the dried gel. Powder XRD analysis of the dried gel was carried out with PANalytical XPERT Pro diffractometer. The X-ray source was Cu Kα radiation (λ = 1.54056 Å) with a voltage of 40 kV, and current of 35 mA. The dried gel was scanned from 1-40° (2θ angle) with step size 0.02°.

11. Dye absorption studies

Organogel (1.5 mL, 1% w/v) was prepared in propan-1-ol in a glass vial (3 nos). Aqueous solution of Direct Red 80 (1.5 mL, 0.02 mM) was added on top of the organogel and this was left undisturbed at room temperature for time-dependent absorption studies (Figure S2a). 1 mL of the dye solution was taken out and used to measure the residual dye concentration by UV–vis spectrophotometer at time points of 6, 12 and 24 h. A standard plot for absorbance (at λ_{max} = 529 nm) versus different dye concentrations was generated (Figure S3a). Using this standard plot, the efficiency of dye absorption by **TPM-G12** gels at different time points was calculated. The same procedure was repeated for crystal violet dye solution (Figure S2b and Figure S3b). λ_{max} of crystal violet dye is 583 nm. We have noted that during the dye absorption studies, the bulk of the gel remained fairly stable during the incubation period. However, the gel-liquid interface tend to be less stable, hence care should be taken during handling of the glass vial.

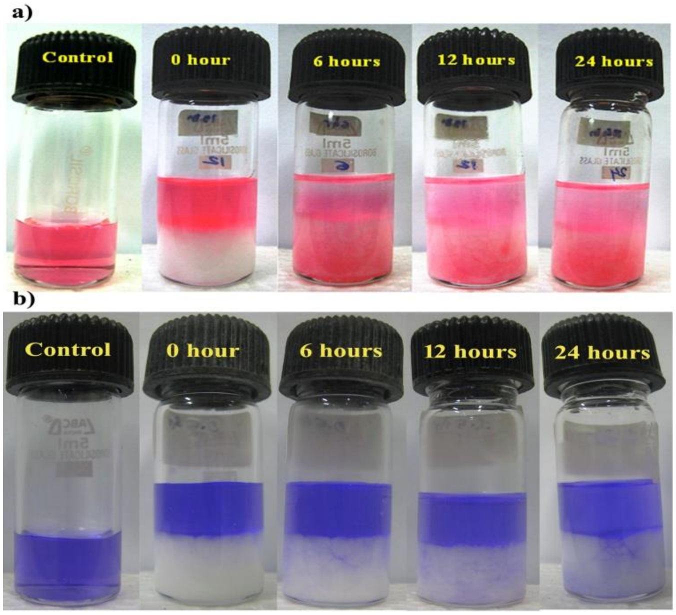

Figure S2: Images for time-dependent absorption of (a) Direct Red 80 (b) Crystal Violet aqueous solution by **TPM-G12** gel (propan-1-ol).

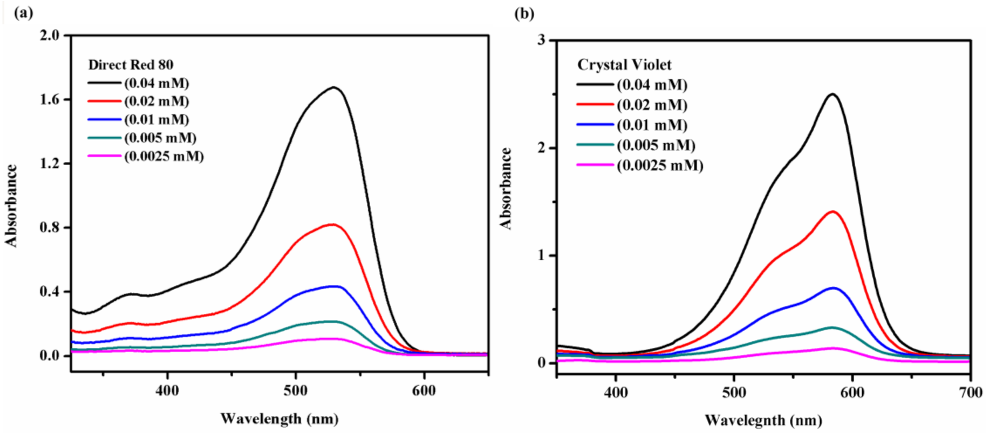

Figure S3: UV–vis absorption profile of (a) Direct Red 80 (b) Crystal violet aqueous solution at different concentrations.

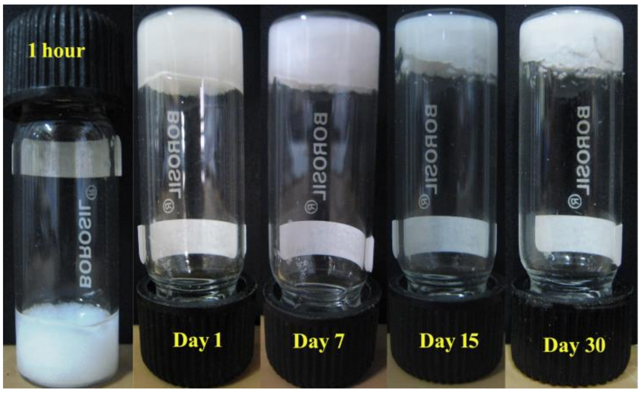

Figure S4: Room temperature stability images for **TPM-G12** gel (1% w/v in propan-1-ol) at different time points.

12. Geometrical optimization method

Geometry optimization of **TPM-G12** and **TPM-G5** was performed using the Gaussian 09 [1] suite of programs. DFT method, the Becke's three-parameter hybrid functional (B3) [2] with the correlation functions of Lee, Yang, and Parr (LYP) [3,4], with basis sets 6-31G (d, p) was used for geometry optimization.

References

1. Frisch, M. J.; Trucks, G. W.; Schlegel, H. B.; Scuseria, G. E.; et. al. Gaussian 09, Revision B.01, Gaussian, Inc., Wallingford CT, **2009**.
2. Becke, A. D. *J. Chem. Phys.*, **1993**, 5648−5652.
3. Lee, C.; Yang, W.; Parr, R. G. *Phys. Rev. B*, **1988**, *37*, 785−789.
4. Miehlich, B.; Savin, A.; Stoll, H.; Preuss, H. *Chem. Phys. Lett.*, **1989**, *157*, 200−206.

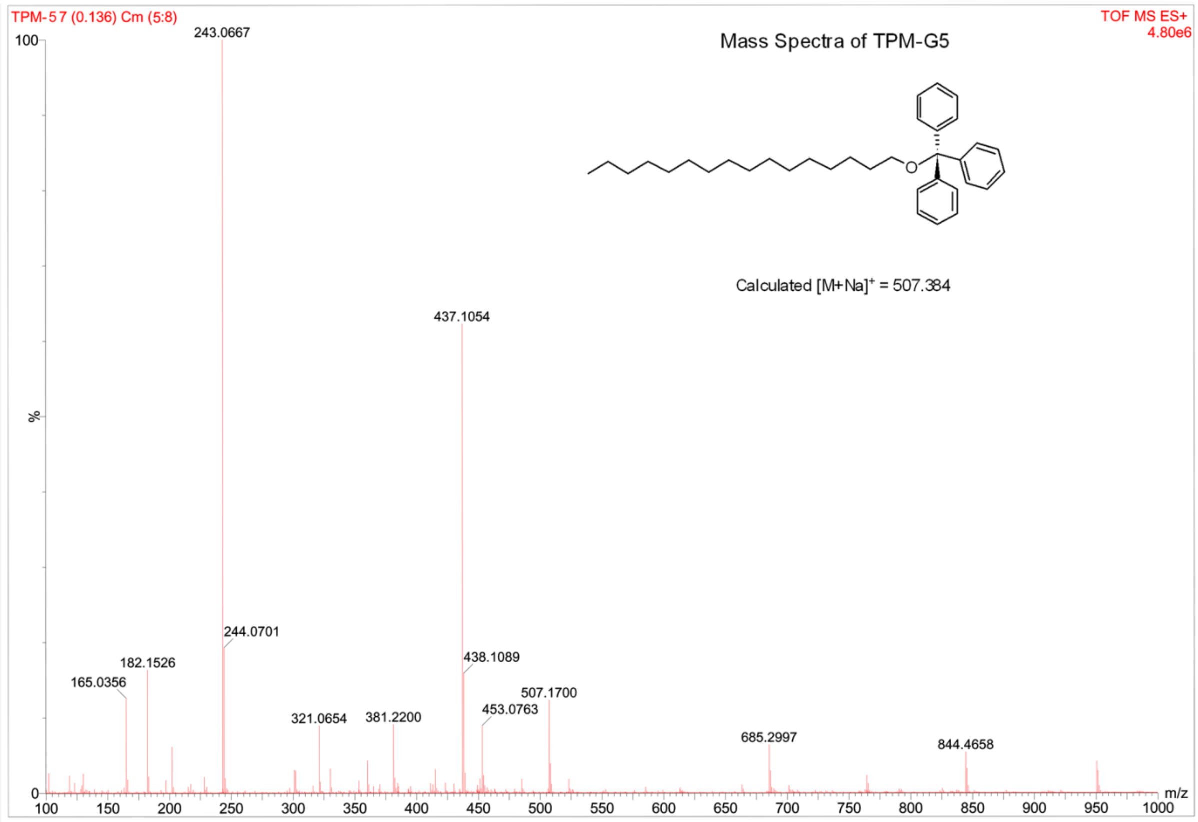

Figure S5: Mass spectra of **TPM-G5**.

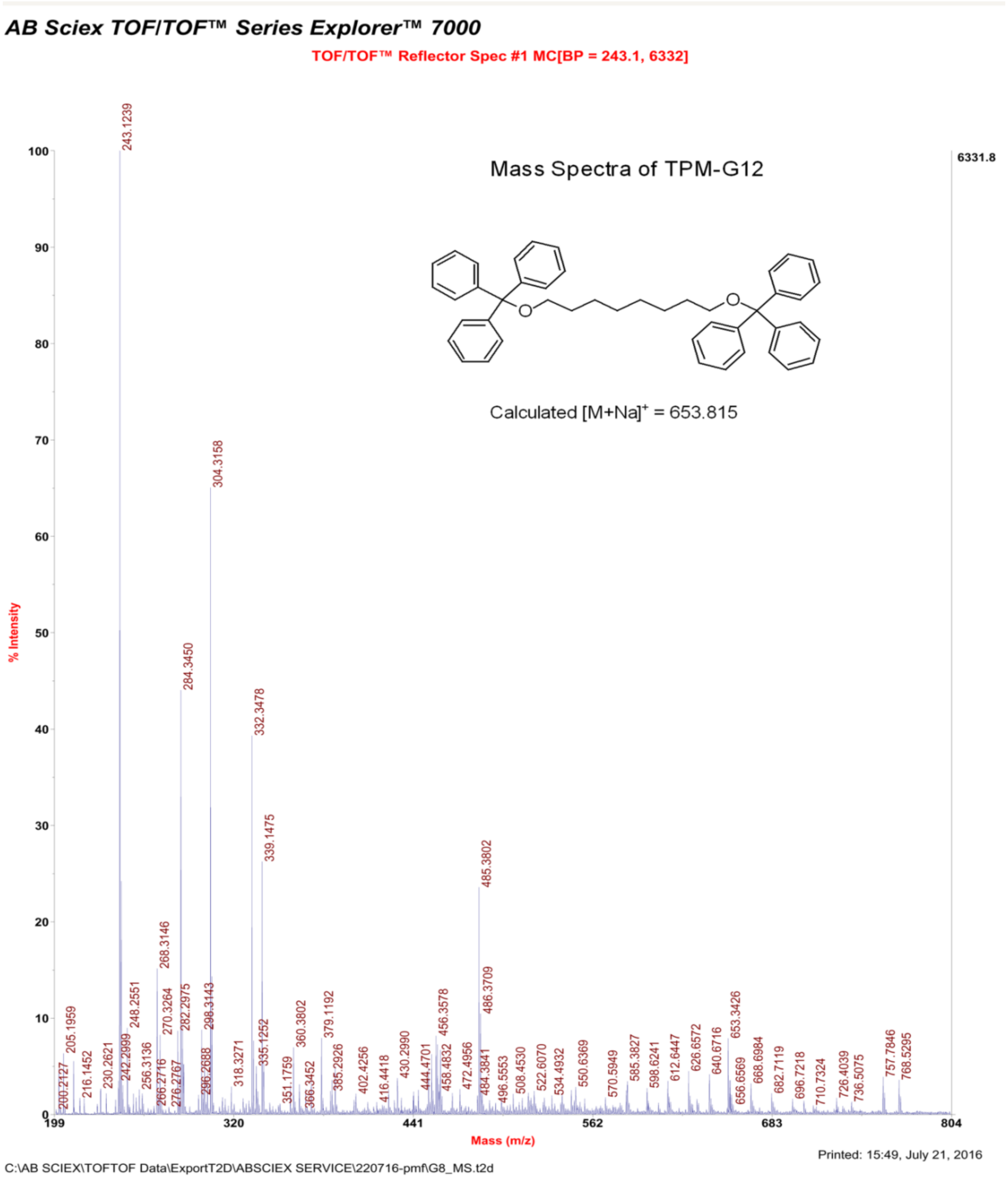

Figure S6: Mass spectra of **TPM-G12**.
